# ChatGPT May Improve Access to Language-Concordant Care for Patients With Non–English Language Preferences

**DOI:** 10.2196/51435

**Published:** 2024-12-10

**Authors:** Fiatsogbe Dzuali, Kira Seiger, Roberto Novoa, Maria Aleshin, Joyce Teng, Jenna Lester, Roxana Daneshjou

**Affiliations:** 1 Department of Dermatology, University of California, San Francisco, 1701 Divisadero Street, San Francisco, CA, 94115, United States, 1 415-353-7800; 2 Department of Dermatology, University of Washington, Seattle, WA, United States; 3 Department of Dermatology, Stanford University, Redwood City, CA, United States; 4 Department of Pathology, Stanford University, Redwood City, CA, United States; 5 Department of Biomedical Data Science, Stanford University, Stanford, CA, United States

**Keywords:** ChatGPT, artificial intelligence, language, translation, health care disparity, natural language model, survey, patient education, preference, human language, language-concordant care

## Abstract

This study evaluated the accuracy of ChatGPT in translating English patient education materials into Spanish, Mandarin, and Russian. While ChatGPT shows promise for translating Spanish and Russian medical information, Mandarin translations require further refinement, highlighting the need for careful review of AI-generated translations before clinical use.

## Introduction

Chat Generative Pre-trained Transformer (ChatGPT) is an artificial intelligence (AI) language model based on natural language processing techniques, developed by OpenAI, that is capable of generating new texts, responding to user input with conversational responses, and summarizing and translating text [1]. The model is trained on large data sets to mimic human language. While it can produce correct responses to queries, ChatGPT is not specifically trained to produce accurate information and may generate false answers that sound plausible.

Prior work evaluating its uses in the medical domain found that ChatGPT could achieve a greater than 60% threshold on NBME (National Board of Medical Examiners) Step 1 questions and provide justification to support most answers [2]. Applications in supporting clinical practice and scientific writing have also been proposed [3]. However, there is a need to explore novel uses to improve patient outcomes and address disparities. Written education materials are critical for communicating information to patients; however, they are not widely available in languages other than English. In 2020, the American Academy of Dermatology website offered information on 6% (5/83) of its listed dermatologic conditions in Spanish [4]. We aimed to evaluate ChatGPT’s ability to translate patient education materials from English to Spanish, Mandarin, and Russian.

## Methods

English-language resources, each fewer than 500 words, used at the University of California, San Francisco, covering 5 dermatology topics, including seborrheic keratosis, sun protection, tinea pedis, warts, and wound care, were translated using the prompt, “Translate this into <target language>.” Excerpts from each topic and the corresponding translations provided by ChatGPT in Mandarin, Spanish, and Russian are shown in Table 1.

**Table 1.** Excerpts from patient educational materials and corresponding translations provided by ChatGPT.

Topic	English **excerpt**	Spanish **translation**	Mandarin **translation**	Russian **translation**
Sun Protection	The sun beams energy to the earth as heat, visible light, and invisible light. Some of the invisible light from the sun called ultraviolet light (\UV light) damages the skin, resulting in:Skin cancers,wrinkling, and premature aging	El sol envía energía a la Tierra en forma de calor, luz visible e luz invisible. Alguna de la luz invisible del sol llamada luz ultravioleta (LUZ UV) daña la piel, lo que resulta en:Cancer de la pielArrugas y envejecimiento prematuro.	太阳将能量以热能, 可见光和不可见光的形式传输到地球。来自太阳的一些不可见光称为紫外线 (UV光），会对皮肤造成伤害，导致：皮肤癌皱纹和过早老化	Солнце передает энергию на Землю в виде тепла, видимого и невидимого света. Некоторый из невидимого света от солнца, называемого ультрафиолетовым светом (UV-свет), наносит вред коже, вызывая:рак кожиморщины и преждевременное старение
Warts	Warts are harmless, noncancerous skin growths caused by a virus. This virus is called the “human papilloma virus” (HPV). The virus makes the top layer of skin grow quickly, causing a wart. The appearance of a wart depends on where it is growing.	Las verrugas son crecimientos de piel inofensivos y no cancerosos causados por un virus. Este virus se llama virus del papiloma humano (VPH). El virus hace que la capa superior de la piel crezca rápidamente, causando una verruga. La apariencia de una verruga depende de dónde esté creciendo.	疣是由病毒引起的无害的, 非癌性的皮肤生长物。这种病毒被称为人乳头瘤病毒 (HPV）。病毒使皮肤的表层迅速生长，从而形成疣。疣的外观取决于其生长的位置。	Бородавки - это безопасные, нераковые росты кожи, вызванные вирусом. Этот вирус называется вирусом папилломы человека (HPV). Вирус заставляет верхний слой кожи расти быстро, что приводит к появлению бородавки. Внешний вид бородавки зависит от того, где она растет.
Wound care dissolvable sutures	Dissolvable sutures, made of materials that the body can break down and absorb, have been used to close your wound. They will not need to be removed. They take approximately 3 weeks to dissolve.	Se han utilizado puntos disueltos, hechos de materiales que el cuerpo puede descomponer y absorber, para cerrar su herida. No será necesario quitarlos. Tardan aproximadamente 3 semanas en disolverse.	可吸收缝线由身体可以分解和吸收的材料制成，用于缝合您的伤口。它们不需要拆除。它们需要大约3周的时间来溶解。	Сухожильные секи из материалов, которые тело может растворить и поглотить, использовались для закрытия раны. Они не нужно удалять. Они растворяются примерно через 3 недели.
Seborrheic keratosis	Treatment options for seborrheic keratoses include:*Freezing or cryosurgery:* The use of liquid nitrogen (a very cold liquid) to destroy the growth. The treated seborrheic keratosis usually falls off days after treatment.	Las opciones de tratamiento para las queratosis seborreicas incluyen: Congelación o criocirugía: el uso de nitrógeno líquido (un líquido muy frío) para destruir el crecimiento. La queratosis seborreica tratada suele caerse días después del tratamiento.	治疗皮脂溢性角化病的选择包括：冷冻或冷冻手术：使用液氮 (一种非常冷的液体）来摧毁生长物。经过治疗的皮脂溢性角化病通常在数天后脱落。	Опции лечения себорейных кератоз: Заморозка или криохирургия: испольжование жидкого азота (очень холодной жидкости) для уничтожения роста. Леченная себорейная кератоза обычно отваливается несколько дней после лечения.
Tinea pedis	Your dermatologist will examine your feet thoroughly. This examination may include a scraping of the scaly area from the skin on your feet. The skin scales are then examined under a microscope to look for the fungus or placed in a test tube to look for growth of the fungus.	Su dermatólogo examinará sus pies a fondo. Este examen puede incluir un raspado de la zona escamosa de la piel de sus pies. Las escamas de la piel luego se examinan bajo un microscopio para buscar el hongo o se colocan en un tubo de ensayo para buscar el crecimiento del hongo.¿Cómo se trata la tinea pedis?	您的皮肤科医生将仔细检查您的双脚。这项检查可能包括从您的足部皮肤上刮取有鳞屑的区域。然后，这些皮肤鳞屑将在显微镜下检查，以寻找真菌或放入试管中以寻找真菌的生长。	Ваш дерматолог будет тщательно осматривать ваши ноги. Это осмотр может включать в себя обрезку шелушащей области с кожи ног. Чешуйки кожи затем исследуются под микроскопом, чтобы искать грибок или помещаются в тест-тубу для поиска роста грибка.

Topics were chosen based on their frequency of discussion in clinics. Resource materials addressed etiology, symptoms, diagnosis, and treatment. In addition, one document addressed expectations and care recommendations for wounds with dissolvable sutures. Board-certified dermatologists fluent in the target languages reviewed the translations for accuracy and suitability for clinical use. One dermatologist was assigned for each target language.

Reviewers completed a 3-question survey indicating the number of instances of misinformation and grammatical errors or misspellings. Misinformation was defined as translation errors that distorted the concept presented in the source document. Reviewers indicated the frequency by selecting one of four options: (1) <3, (2) 3‐5, (3) 6‐10, and (4) >10. Reviewers also indicated their level of agreement with the statement, “I could provide this to a patient speaking this language,” on a 5-point Likert scale: (1) strongly agree, (2) agree, (3) neutral, (4) disagree, and (5) strongly disagree.

### Ethical Considerations

The study adhered to institutional ethical guidelines. The study did not require approval from an institutional review board or research ethics board because the study did not involve identifiable private information or clinical intervention involving humans subjects. Informed consent was not applicable for this study.

## Results

The results of the survey are shown in Table 2. Misinformation was prevalent in materials translated into Mandarin, with 60% (3/5) of translations containing between 3 and 5 instances. Grammatical and spelling errors were identified for all languages. For Mandarin, 60% (3/5) had 6-10 errors, and 40% (2/5) had between 1 and 5 errors. For Russian, 20% (1/5) had 6-10 errors, and 80% (4/5) had more than 10 errors. For Spanish, 40% (2/5) documents had 3-5 errors, and 60% (3/5) had 6-10 errors. When asked whether they would provide the translation to patients, the reviewers responded “agree” for 80% (4/5) of Russian and Spanish documents and 20% (1/5) of Mandarin documents. Moreover, 60% (3/5) of Mandarin documents received a “disagree” response.

**Table 2.** Responses to survey evaluating misinformation, number of errors, and usability of ChatGPT translations.

Target language and topic	Instances of misinformation	Grammatical and spelling errors	Rate this statement: I could provide this to a patient speaking this language
Mandarin
Seborrheic keratosis	3‐5	6‐10	Disagree
Sun protection	<3	<3	Agree
Tinea pedis	3‐5	6‐10	Disagree
Warts	3‐5	6‐10	Disagree
Wound care	<3	3‐5	Neutral
Spanish
Seborrheic keratosis	<3	6‐10	Agree
Sun protection	None	3‐5	Agree
Tinea pedis	None	6‐10	Agree
Warts	<3	3‐5	Neutral
Wound care	None	6‐10	Agree
Russian
Seborrheic keratosis	None	6‐10	Agree
Sun protection	None	>10	Agree
Tinea pedis	None	>10	Agree
Warts	None	>10	Agree
Wound care	<3	6‐10	Disagree

## Discussion

This study’s findings indicate that ChatGPT may be an effective tool for translating patient education materials into Spanish and Russian. Despite some errors, most translations were deemed clinically usable. Mandarin translations, however, were less reliable and deemed insufficient for patient use. ChatGPT may be a promising tool for expanding access to language-concordant care; however, further investigation is necessary, given that its proficiency appears to vary by language.

Previous studies have demonstrated ChatGPT’s ability to answer medical licensing examination questions in Spanish and Chinese, underscoring its potential applications in medical education in non–English languages [5,6]. However, one study concluded that its performance in Chinese is suboptimal for medical education and clinical decision-making compared to English [7]. The linguistic complexity of Mandarin poses challenges; differences in sentence structure and terminology between Mandarin and English likely contribute to this suboptimal performance, highlighting the need for caution when using AI translation for languages with significant linguistic differences from English.

Our study is limited by its focus on dermatologists’ expertise for evaluating translations and the use of existing materials without exploring multiple prompts, thus potentially underestimating the effects of errors on comprehension and credibility. Ultimately, any implementation of translated documents should be preceded by careful proofreading to ensure their suitability given variable efficacy by language.

## Abbreviations

AI: artificial intelligence
ChatGPT: Chat Generative Pre-training Transformer
NBME: National Board of Medical Examiners
